# Dataset for evaluation and numerical modelling of structural performance of fibre-reinforced shotcrete with fibres of steel, synthetic and basalt

**DOI:** 10.1016/j.dib.2025.111684

**Published:** 2025-05-18

**Authors:** Andreas Sjölander, Erik Nordström, Anders Ansell

**Affiliations:** a KTH Royal Institute of Technology, Division of Concrete Structures, Brinellvägen 23, 114 28, Stockholm, Sweden; b Vattenfall R&D, Älvkarlebylaboratoriet, 814 70, Älvkarleby, Sweden

**Keywords:** Round determinate panel, Beam testing, Calibration of material models, Residual flexural strength, Energy absorption

## Abstract

This dataset [1] contains results from structural testing of cast and sprayed fibre-reinforced shotcrete with steel, synthetic and basalt fibres. This includes testing residual flexural strength on beams according to EN 14488-3 [2] and the energy absorption of round determinate panels tested according to ASTM C1550 [3]. For all tests, the compressive strength was evaluated according to EN 12390-3 [4]. Four different fibre types were tested, and for each fibre type, three dosages were tested. Beams were produced by casting, while panels were produced by casting and spraying. This resulted in a total of 36 beams and 72 panels and cubes. Structural testing was performed at an accredited laboratory by certified personnel using standard equipment found in a commercial laboratory for structural testing.

The dataset contains a summary of the standard parameters evaluated for each test method but also contains the raw data from the test machine for tests on beams on panels. One unique property of this dataset is the variation in fibre types, i.e. testing includes steel, synthetic and basalt fibres. The standard test parameters summary is useful for directly comparing and evaluating the structural performance for each fibre type and dosage. This could be used to get an indication of the required dosage of each fibre type to fulfil structural requirements. The raw data from the test machine is valuable for the numerical modelling of fibre-reinforced shotcrete. This data contains the relationship between external force and vertical displacement of the specimen and can be used to calibrate a material model for finite element simulations.

Specifications Table

**Table:** 

Subject	Civil and Structural Engineering
Specific subject area	Testing of flexural residual strength and energy absorption of fibre-reinforced shotcrete with steel, synthetic and basalt fibres
Type of data	Raw data in .txt file Filtered data in .csv and .pickle Summarised data in table format (xlsx format), Figure plot of data (.jpg), Python code for data analysis
Data collection	Data was collected during experimental testing using standard equipment for structural testing of concrete available in commercial laboratories. Testing was performed by accredited personnel. Specifically, an MTS test rig was used.
Data source location	Data was obtained at Vattenfall R&D in Älvkarleby, Sweden and is stored on Mendeley Data.
Data accessibility	Repository name: Mendeley Data Data identification number: 10.17632/d7n5mvb2sg.2 Direct URL to data: https://data.mendeley.com/datasets/d7n5mvb2sg/2
Related research article	None

## 1 Value of the Data

 

• This dataset gives valuable insight into the structural performance of steel, synthetic and basalt fibre-reinforced shotcrete. Testing was done using the same shotcrete mix, which enables a direct comparison between the performances of different fibre types. For owners or designers of structures with fibre-reinforced shotcrete, this gives valuable information regarding the dosage of fibres required to achieve a target flexural strength or energy absorption. Thus, the data can be used for preliminary design and comparison between which fibre type that is suitable to use.
• The dataset provides an unique test series, in which the structural performance of four different fibre types have been tested using two commonly used standard methods. The dataset contains both cast and sprayed specimens. In the literature, cast samples are commonly used and this data can therefore be valuable for both researches and designers to estimate the effect of the production method.
• For the research community focusing on structural performance of fibre-reinforced concrete, the dataset can be used to create finite element models of fibre-reinforced concrete. In particular, the dataset can be used to calibrate material models for different fibre types. The presented data from the test machines can be used to compare the response between experimental testing and numerical simulations. This type of data is seldom published in the literature.
• For the design of fibre-reinforced shotcrete, establishing a correlation between flexural residual strength measured on beams and energy absorption measured on panels have the potential to lower the required dosages of fibres. With data available from two test methods, i.e. beams and panels, this dataset increases the number of available data to further studies on the potential correlation between residual flexural strength measured on beams and energy absorption measured on panels.

## 2 Background

This data was collected during a project focusing on the feasibility of using synthetic and basalt fibres as an alternative to steel fibres to reinforce shotcrete used for tunnel support. The first dataset was published in 2022 [1] and used to highlight how the design philosophy and test methodology influence the required amount of fibres used in shotcrete [5]. In 2025, the original dataset [1] was updated with the results from sprayed samples. The reason for the large experimental campaign was the lack of available datasets in the literature. Although experimental testing of fibre-reinforced concrete and shotcrete is commonly published, authors seldom make all results from the experiments available. Moreover, most research focuses on one type of fibre, e.g. steel or synthetic. Since the post-cracking behaviour of fibre-reinforced shotcrete depends on the pull-out of individual fibres, the interaction between fibres and shotcrete is crucial. Thus, the material mix is essential, and comparing tests performed with different shotcrete mixes is difficult. Therefore, the same shotcrete mix was used throughout the entire test series, and the only variables were the fibre type and dosage.

## 3 Data Description

The dataset is available on Mendeley Data [1] and is structured in seven main folders. The content is described below.

### 3.1 ASTM C1550

This folder contains the raw data from testing of energy absorption of round determinate panels according to the test standard ASTM C1550 [3]. The folder contains one folder for cast and one for sprayed data. Each of these folders contains one subfolder for each fibre type and dosage and is named accordingly, e.g. “Barchip 54 3 kg” and “Dramix 3D 30 kg”. Each folder contains three .txt files with the raw data from each test. Each file contains three columns of data with the following data:

1 = Load (kN) – 2 = Disp. (um) and – 3 = Time (s).

The columns are separated by “,” and “.” is used as decimal.

### 3.2 Data

This folder contains the folders “Filtered” and “Raw”. The “Raw” folder contains an Excel spreadsheet summarizing all the key testing results. The spreadsheet contains the following sheets:

• Mix: Description of the shotcrete mix
• Cube-C: Compressive strength results for cast cubes according to EN 12390-3 [4].
• Cube-S: Compressive strength results for drilled cores according to EN 12390-3 [4]. Cores are from sprayed panels after completion of the test according to ASTM C1550 [3].
• Beams-C: Tensile strength at cracking and flexural residual strength values $f_{0.5}$ - $f_{5,}$ which are values defined by the standard EN 14488-3 [2]
• Panels-C: Force and displacement at cracking and the energy absorption from tests on cast panels according to ASTM C1550 [3] based on values defined in the standard.
• Panels-S: Same as above but for sprayed samples.

The “Filtered” folder contains the data from the testing of cubes, beams and panels in .csv format and in the format pickle, which is a Python object. These files can be read and imported using Python, Excel, Matlab, or any other suitable program. The folder also contains the file fibre pullout pickle, which contains the data from testing the fibre pullout stored in Python dictionary format. This data can be imported using Python and the library Pandas (pd) with the command “pd.readpickle(filename).”

### 3.3 EN 14488-3

This folder contains the data from cast beams tested according to EN 14488-3 [2]. It contains the folder Cast Samples, which contains one subfolder for each fibre type and dosage. Each subfolder contains three .txt files with the raw data from the test machine. The file contains two columns: the first is the vertical displacement given in mm, and the second column is the load in N. The columns are separated by “,” and “.” is used as decimal.

### 3.4 Plot

This folder contains the following four subfolders:

• Cast Beams: Contains .jpg images with plotted results for force and displacement for tests on cast beams. One plot for every tested fibre type and dosage, i.e. 12 plots in total.
• Cast Panels: Contains .jpg images with plotted results for force and displacement for cast panels. One plot for every tested fibre type and dosage, i.e. 12 plots in total.
• Pullout: Contains .jpg images with plotted results for force and displacement for pullout tests performed using four different fibres. One plot for every tested fibre type, i.e. four plots in total.
• Sprayed panels: Contains .jpg images with plotted results for force and displacement for sprayed panels. One plot for every tested fibre type and dosage, i.e. 12 plots in total.

### 3.5 Pull-out

This folder contains results from pullout tests of individual fibres. Here, four different fibres were used, and the original data is stored in .xlsx format. In each spreadsheet, three columns with data are available: Time (s), Axial Force (N) and Axial Displacement (mm). The data is arranged in one subfolder for every fibre type and one folder containing all the data.

### 3.6 Python

This folder contains Python scripts for basic operations such as importing, filtering, grouping and plotting the data. All scripts are made with relative links and should, therefore, work if the original folder structure of the dataset is contained on your local computer. The file “Info.py” contains a brief explanation of each of the Python scripts.

## 4 Experimental Design, Materials and Methods

Below, an overview of the experimental campaign is first presented. Thereafter, the production of the specimens is explained, followed by a description of the test methods.

### 4.1 Overview of the experimental campaign

An overview of the experimental campaign is presented in Table 1. The dataset contains a total of 72 tests on compressive strength and energy absorption and 36 tests on residual flexural strength.

**Table 1:** Overview of experimental campaign on fibre-reinforced shotcrete.

Fibre	Material	Dosage (kg/m^3^)	Cast			Sprayed	
			Cubes (-)	Beams (-)	Panels (-)	Cores (-)	Panels (-)
Dramix 3D	Steel	30 / 40 / 50	3 / 3 / 3	3 / 3 / 3	3 / 3 / 3	3 / 3 / 3	3 / 3 / 3
Dramix 4D	Steel	20 / 30 / 50	3 / 3 / 3	3 / 3 / 3	3 / 3 / 3	3 / 3 / 3	3 / 3 / 3
Minibar	Basalt	14 / 16 / 20	3 / 3 / 3	3 / 3 / 3	3 / 3 / 3	3 / 3 / 3	3 / 3 / 3
Barchip 54	Synthetic	3 / 6 / 9	3 / 3 / 3	3 / 3 / 3	3 / 3 / 3	3 / 3 / 3	3 / 3 / 3

The mechanical properties of the tested fibres and the shotcrete mix are presented in Table 2, Table 3, respectively.

**Table 2:** Mechanical properties of tested fibres.

Fibre	Density (kg/m^3^)	E (GPa)	$f_{u}$ (MPa)	l (mm)	d (mm)	l/d (-)
Dramix 3D	7850	200	1800	35	0.55	65
Dramix 4D	7850	200	1600	35	0.55	65
Minibar	2000	42	> 1000	43	0.7	61
Barchip 54	900	12	640	54	0.6	90

**Table 3:** Shotcrete mix.

Material	Quantity	Unit
Cement	500	kg/m^3^
Water	205	kg/m^3^
w / c	.41	-
Aggregate 0 - 2 mm	476	kg/m^3^
Aggregate 0 - 8 mm	1111	kg/m^3^
Release agent^1^	1.6	kg/m^3^
Plasticizer^2^	5.0	kg/m^3^
Air^3^	4.5 – 5.1	%
Slump^3^	80 – 160	mm

1 Sika Perfin 301.

2 Sika Visco-Crete 6730.

3 Given as range for all samples.

### 4.2 Preparation of test samples

The shotcrete was mixed using a rotating mixer with a sufficient capacity to create all specimens from one batch of shotcrete, i.e. three cubes, beams and panels were cast from one batch. Steel moulds were used for cubes and panels, while beams were prepared according to EN 12390-1 [6]. Cast shotcrete specimens were produced by placing shotcrete in the mould, then vibrating and levelling to ensure a smooth surface. To produce sprayed specimens, the shotcrete was after mixing placed in a spraying robot, and panels were sprayed with a handheld spray robot. Mixing and casting of specimens were done by certified laboratory personnel at Vattenfall R&D, while spraying was performed by certified spray operators from the contractor BESAB.

**Fig. 1:**
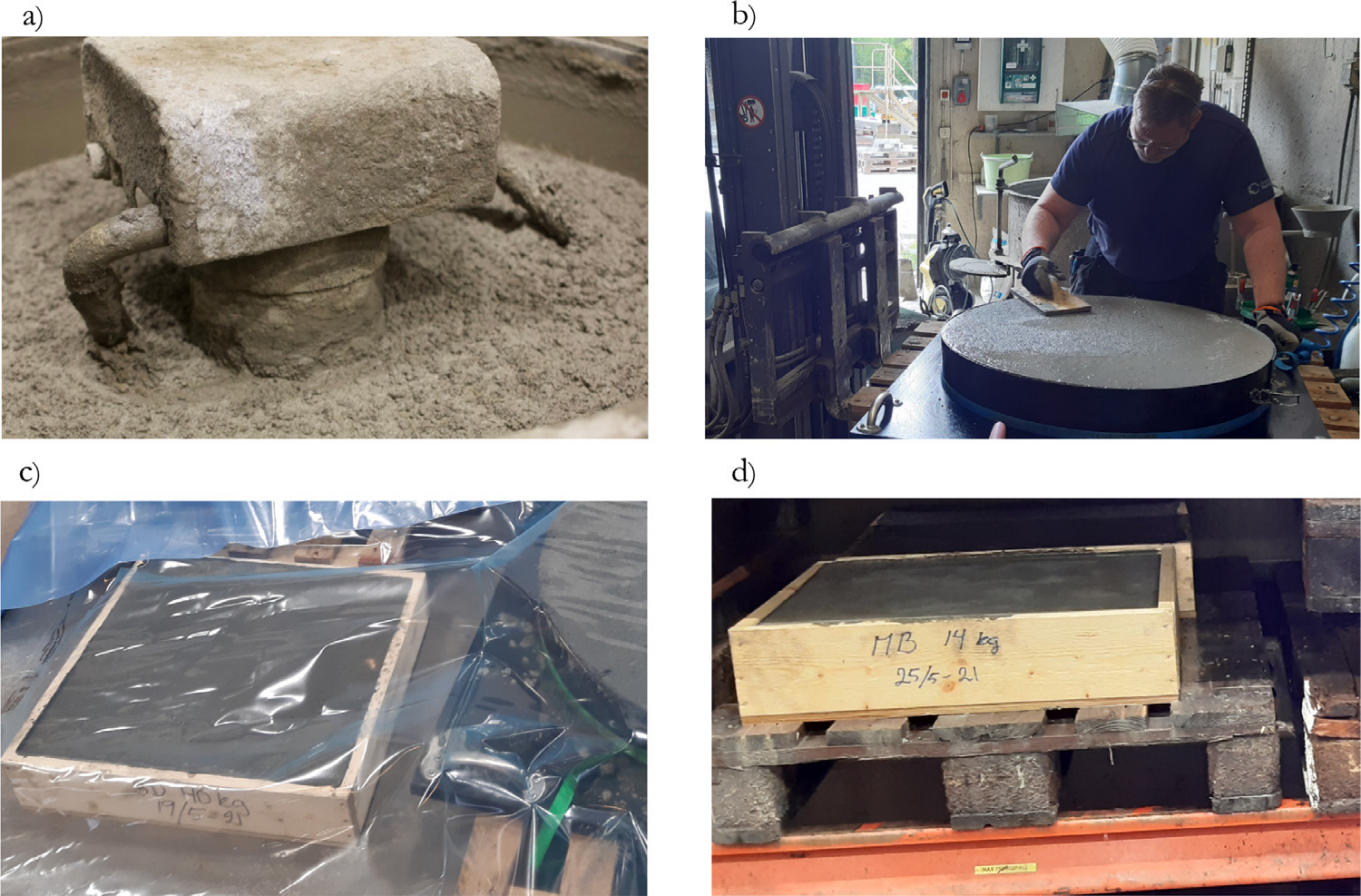
Showing mixing of cast shotcrete a), preparation of cast specimens b), curing during first 24 hours c) and storing and curing before testing d).

Directly after casting and spraying, the samples were covered with plastic sheets for the first 24 hours. Thereafter, the samples were stored in an indoor climate where water was added regularly. After 21 days of hardening, standard beams were cut from the panels and testing was performed after a minimum of 28 days of hardening. The preparation process is shown in Fig. 1.

### 4.3 Testing of compressive strength

The compressive strength was evaluated for every concrete batch as a reference for the quality of the material. Tests were performed in accordance with EN 12390-3 [4]. For cast specimens, 150 mm cubes were used. For sprayed specimens, 65 mm cores were drilled out from the panels after the testing of energy absorption was complete.

### 4.4 Testing of flexural residual strength

The residual flexural strength was evaluated based on EN 14488-3 [2]. According to this standard, a 75 × 125 × 500 mm (Height × Width × Length) beam is tested in four-point bending until a minimum vertical deformation of 4 mm is achieved. The vertical displacement is measured at the centre of the beam using two LVDT mounted on each side of the beam. The displacement presented in the data is the mean value of these two. The preparation of the beams should follow EN 12390-1 [6] and include casting a panel from which the beams are later sawn out. The residual flexural strengths $f_{r1}$-$f_{r5}$ are calculated as follows:

• $f_{r1}$ is calculated based on the minimum load between 0.5 to 1.0 mm deflection
• $f_{r2}$ is calculated based on the minimum load between 0.5 to 2.0 mm deflection
• $f_{r3}$ is calculated based on the minimum load between 0.5 to 3.0 mm deflection
• $f_{r4}$ is calculated based on the minimum load between 0.5 to 4.0 mm deflection
• $f_{r5}$ is calculated based on the minimum load between 0.5 to 5.0 mm deflection

**Fig. 2:**
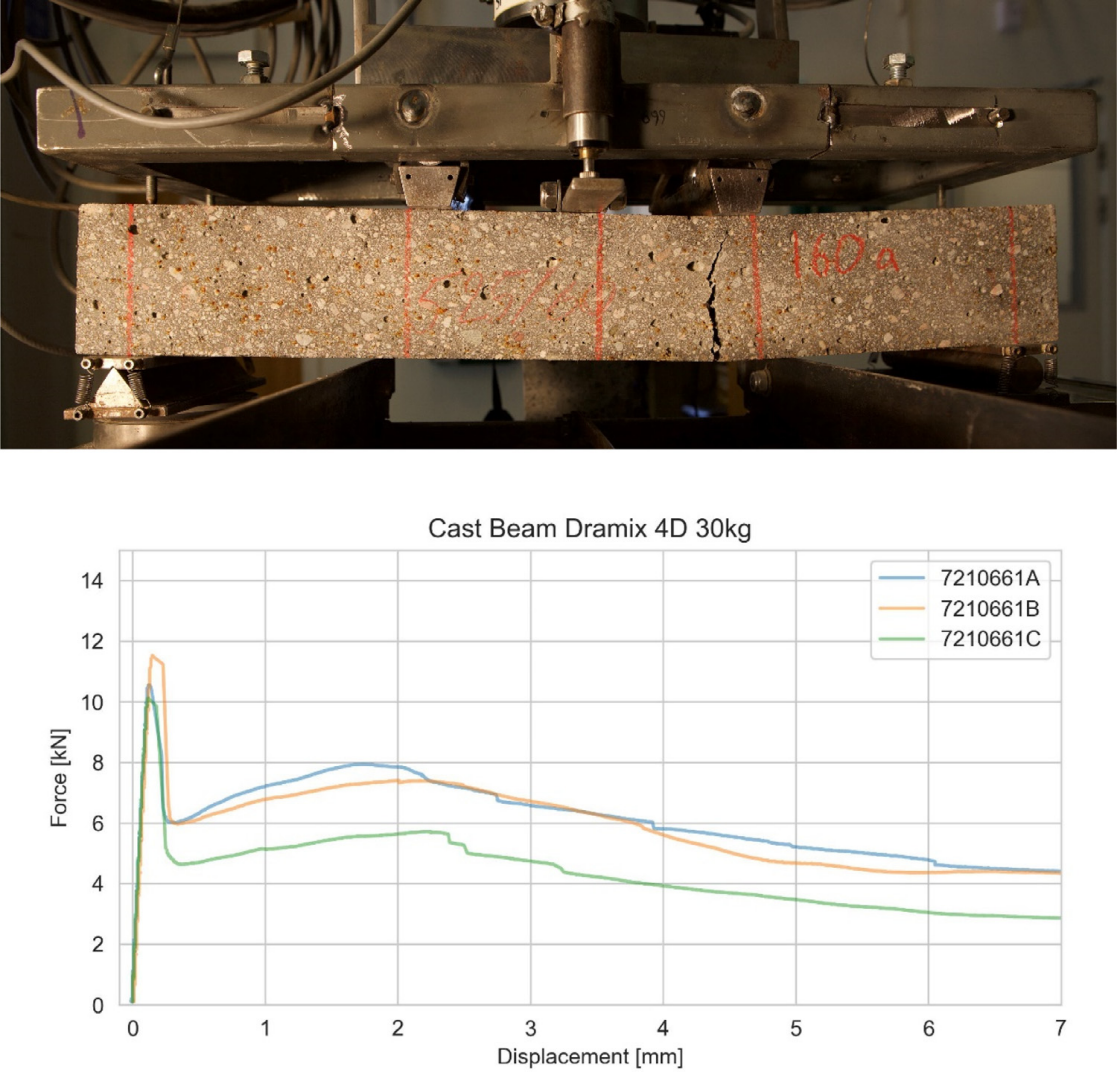
Testing of fibre-reinforced shotcrete according to the standard EN 14488-3 [2] showing set-up (top) and example of results (bottom).

Besides the residual strengths, the load and displacement at cracking are also noted. A figure showing the test setup and example of test results are shown in Fig. 2.

### 4.5 Testing of energy absorption

**Fig. 3:**
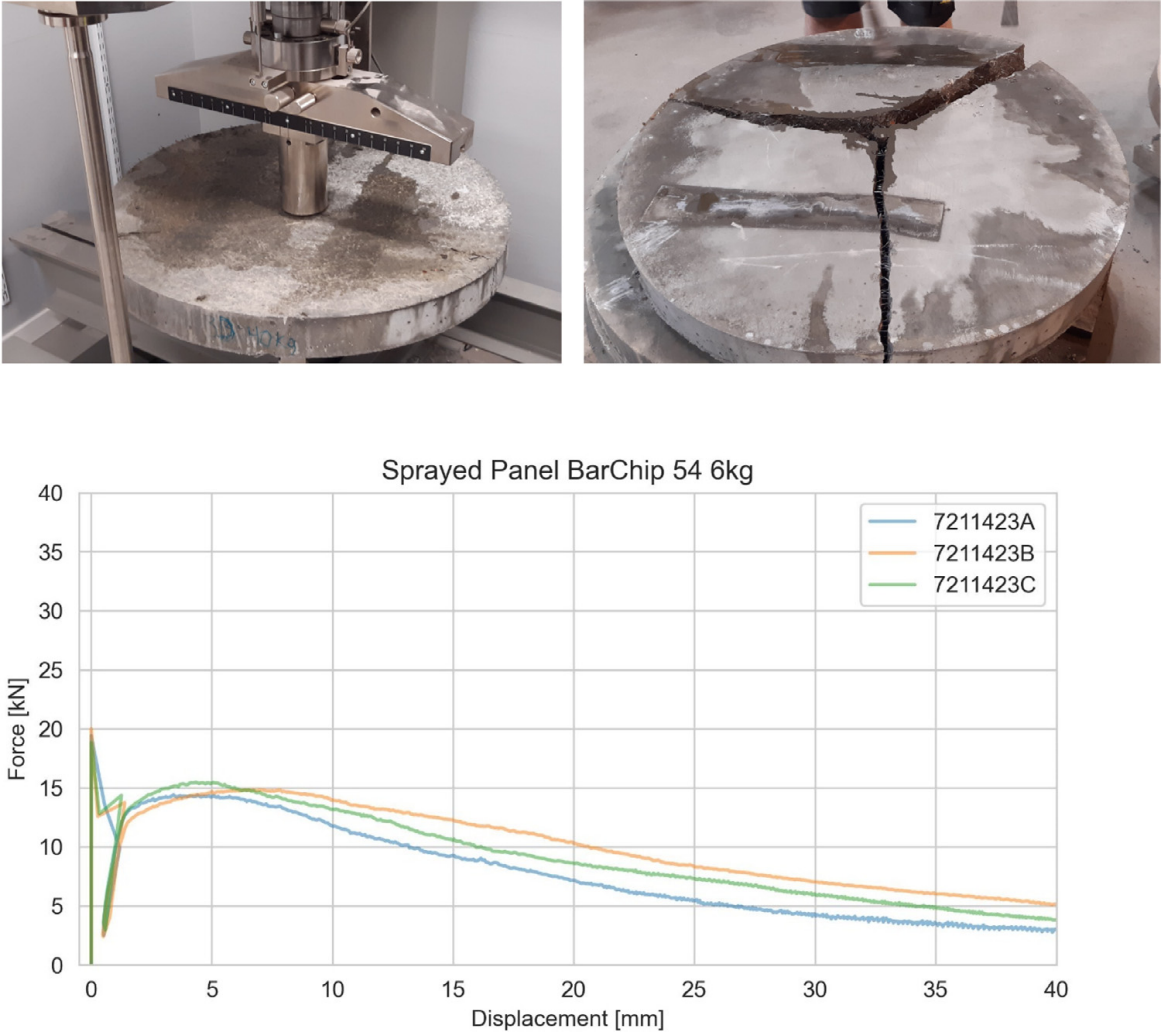
Testing of round determinate panel and cracking after completion of test shown in the top figure and examples of test results (bottom).

Energy absorption for cast and sprayed panels was tested on round determinate panels according to ASTM C1550 [3]. The panel has a diameter of 800 mm and a thickness of 75 mm. It is prepared by spraying or casting shotcrete directly into a steel mould. Correct thickness is achieved by levelling the specimen directly after casting or spraying. During testing, the plate is supported on three pivot points that allow rotation of the panel fragment after cracking [3], and a successful test should yield three radial cracks. The displacement is measured with a single LVDT mounted under the centre of the slab. The test is terminated at a vertical deformation of 40 mm, and the energy absorption, i.e. the area under the load-displacement diagram is calculated at 5, 10, 20, 30 and 40 mm vertical deflection. The test setup and a successfully tested panel are shown in Fig. 3.

### 4.6 Testing of pull-out

**Fig. 4:**
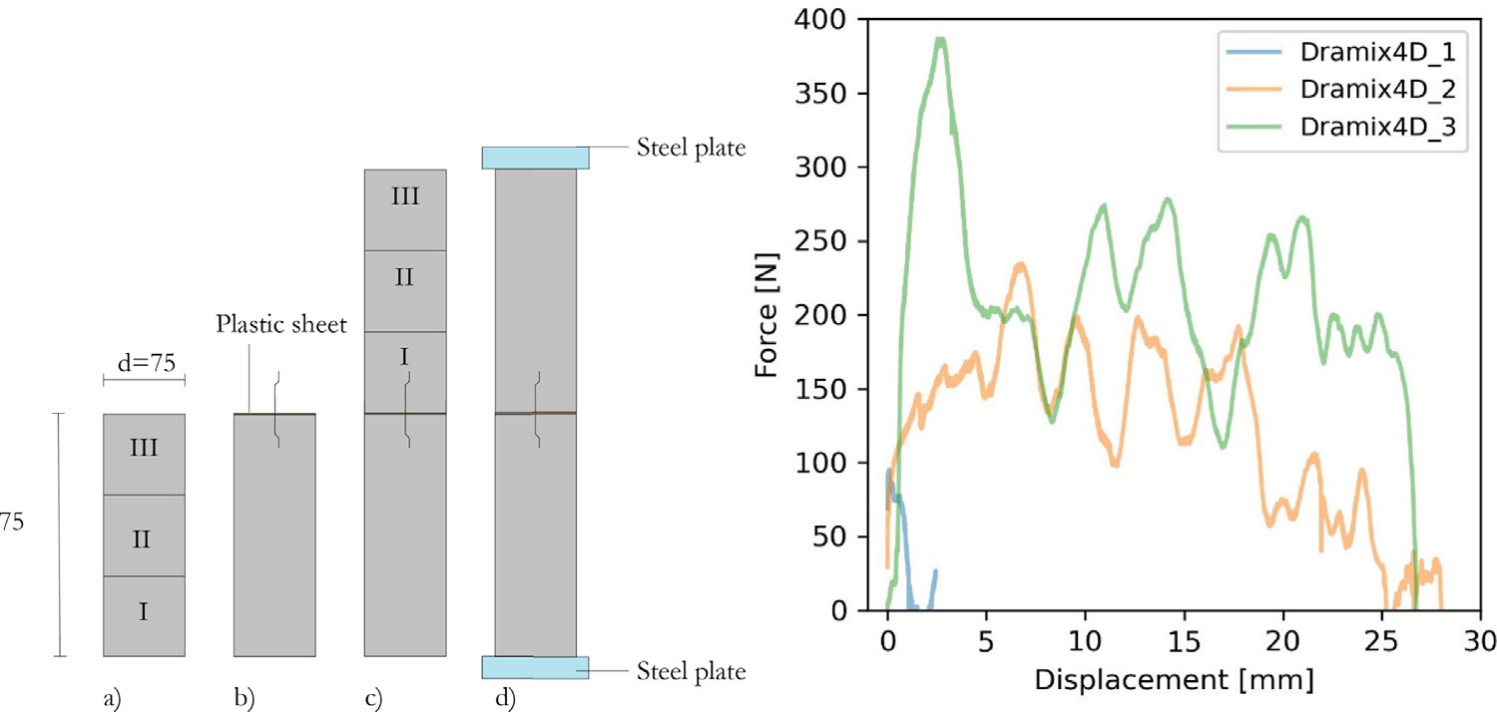
Preparation and set-up for testing of pull-out of individual fibres (left) and examples of results from testing of Dramix 4D fibres. From Sjölander et al. [7].

Pull-out tests on individual fibres were performed using two concrete cylinders conencted with a single fibre as shown in Fig. 4. The height and diameter of each cylinder was 75 mm. Each cylinder were produced by filling shotcrete in three layers. After each layer, the shotcrete was compacted using a steel rod. After filling the first cylinder, a single fibre was embedded with half its length in the shotcrete. A plastic sheet was then placed on the surface to prevent bond between to the second concrete cylinder. The second cylinder was cast on top of the first cylinder using the same principle as for the first cylinder. The joint between the cylinders was covered with tape and the specimens were covered with plastic and cured indoors with an approximate temperature of 20 ˚C. During testing, steel plates were glued to the specimens which then were subjected to uniaxial tension until failure.

## Limitations

One panel test was stopped prematurely. This is noted with a “-“ in the raw data spreadsheet.

## Ethics Statement

The authors confirm that they have read and followed the ethical requirements for publication in Data in Brief and confirm that the current work does not involve human subjects, animal experiments, or any data collected from social media platforms.

## CRediT Author Statement

**Andreas Sjölander:** Writing - Original draft preparation, Data curation, Experimental planning, Investigation. **Erik Nordström:** Writing – Reviewing and Editing, Experimental planning. **Anders Ansell:** Writing – Reviewing and Editing, Experimental planning.

## Data Availability

Mendeley DataData from structural testing of sprayed and cast shotcrete reinforced with fibres of steel, basalt and synthetic material (Original data)
